# Single-cell assessment of transcriptome alterations induced by Scriptaid in early differentiated human haematopoietic progenitors during *ex vivo* expansion

**DOI:** 10.1038/s41598-019-41803-z

**Published:** 2019-03-28

**Authors:** Peng Hua, Barbara Kronsteiner, Mark van der Garde, Neil Ashley, Diana Hernandez, Marina Tarunina, Lilian Hook, Yen Choo, Irene Roberts, Adam Mead, Suzanne M. Watt

**Affiliations:** 1 MRC Molecular Haematology Unit, Weatherall Institute of Molecular Medicine, Radcliffe Department of Medicine, University of Oxford, John Radcliffe Hospital, Oxford, OX3 9DS UK; 2 Stem Cell Research, Nuffield Division of Clinical Laboratory Sciences, Radcliffe Department of Medicine, University of Oxford, and NHS Blood and Transplant, John Radcliffe Hospital, Oxford, OX3 9BQ UK; 3 Plasticell Ltd, Stevenage Bioscience Catalyst, Stevenage, SG1 2FX UK; 4 Department of Paediatrics, University of Oxford, Children’s Hospital, John Radcliffe Hospital, Oxford, OX3 9DU UK; 5 Haematology Theme, Oxford Biomedical Research Centre, Oxford University Hospitals, Oxford, UK

## Abstract

Priming haematopoietic stem/progenitor cells (HSPCs) *in vitro* with specific chromatin modifying agents and cytokines under serum-free-conditions significantly enhances engraftable HSC numbers. We extend these studies by culturing human CD133+ HSPCs on nanofibre scaffolds to mimic the niche for 5-days with the HDAC inhibitor Scriptaid and cytokines. Scriptaid increases absolute Lin−CD34+CD38−CD45RA−CD90+CD49f+ HSPC numbers, while concomitantly decreasing the Lin−CD38−CD34+CD45RA−CD90− subset. Hypothesising that Scriptaid plus cytokines expands the CD90+ subset without differentiation and upregulates CD90 on CD90− cells, we sorted, then cultured Lin−CD34+CD38−CD45RA−CD90− cells with Scriptaid and cytokines. Within 2-days and for at least 5-days, most CD90− cells became CD90+. There was no significant difference in the transcriptomic profile, by RNAsequencing, between cytokine-expanded and purified Lin−CD34+CD38−CD45RA−CD49f+CD90+ cells in the presence or absence of Scriptaid, suggesting that Scriptaid maintains stem cell gene expression programs despite expansion in HSC numbers. Supporting this, 50 genes were significantly differentially expressed between CD90+ and CD90− Lin−CD34+CD38−CD45RA−CD49f+ subsets in Scriptaid-cytokine- and cytokine only-expansion conditions. Thus, Scriptaid treatment of CD133+ cells may be a useful approach to expanding the absolute number of CD90+ HSC, without losing their stem cell characteristics, both through direct effects on HSC and potentially also conversion of their immediate CD90− progeny into CD90+ HSC.

## Introduction

Haematopoietic stem cells (HSCs) are used clinically to treat severe blood diseases^1^ or generate mature effector-cells for transfusion^2^, while precision genome editing combined with HSC transplantation may cure certain blood and immune disorders (e.g. haemoglobinopathies, HIV-AIDS, SCID-X1)^2–5^. Culture conditions, which increase HSC numbers or promote HSC cycling for effective gene editing^6^ without compromising their stem cell characteristics, would enhance their therapeutic applicability.

Epigenetic mechanisms are important in regulating HSC fate^7–11^. Combining histone deacetylase inhibitors (HDACi) with cytokine priming under serum-free conditions can significantly enhance expansion *ex vivo* of Lin−CD34+CD38−CD45RA−CD90+CD49f+ early HSPCs and/or NSG-engraftable human cord blood (UCB) HSC (SCID repopulating cells or SRC)^12^. This has been shown to be dependent on the specific HDACi used. Various researchers have demonstrated that HDACis, such as Valproic acid (VPA), Scriptaid (Scr), Trichostatin (TSA), Suberoylanilide hydroxamic acid (SAHA or Vorinostat), CAY10433, CAY10398 and CAY10603 allow greater expansion of UCB CD34+, CD34+CD90+ HSPC and/or early *in vitro* clonogenic cobblestone area forming cells (CAFC) or long term culture-initiating cells (LTC-IC) in *ex vivo* short term (up to 9 days) cultures in the presence of cytokines than with cytokines alone^12–19^. Of these, three class I/II HDCAis, VPA, Scriptaid and CAY10433 are reported to generate, albeit to differing degrees, higher absolute numbers of UCB CD34+ and CD34+CD90+ HSPCs when added individually to serum-free cultures with stem cell factor (SCF), Flt-3 ligand (FL), thrombopoietin (TPO) and interleukin-3 (IL-3) for 7 days^12^. Interestingly, both VPA^12,18^ or Scriptaid (as presented here) addition to cytokine-driven cultures significantly increases the absolute numbers of HSPCs expressing Lin−CD34+CD38−CD45RA−CD90+CD49f+ biomarkers, which define the main phenotype of uncultured HSCs. In surrogate transplant models, greater frequencies of human CD45+ cell engraftment into the bone marrow of transplanted primary NSG immunodeficient mice (e.g. 100% vs 20% of mice with 2,500 culture initiating cell equivalents infused) and greater degrees of human CD45+ cell chimaerism (on average 2.4 fold higher) at weeks 12–14 post transplant were also observed when human UCB HSPC expanded in VPA with cytokines for 7 days were compared to those expanded with cytokines alone^12,18^. We have also carried out preliminary *in vivo* repopulation experiments of UCB CD133+ HSPCs expanded in Scriptaid and SCF, TPO and FL cytokines versus these cytokines alone for 5 days on nanofibre scaffolds (the cultures being supplemented with these factors at, and 2 days after, the beginning of the cultures). At week 16 post transplant, we observed a greater frequency of engraftment with the Scriptaid plus cytokine cultured cells as opposed to cytokine alone cultured cells (e.g. 100% vs 40% engrafting respectively into 3 and 5 NSG mice with infusion of 2,500 culture initiating CD133+ cell equivalents) and greater degrees of human CD45+ cell chimaerism (on average 3.6 fold higher; Watt SM *et al*. unpublished data). Higher *in vivo* primary NOD/SCID engraftment of human CD34+ cells was also observed with the sequential addition of 5-azacytidine followed by TSA in the presence of cytokines (SCF, TPO, FL) than with cytokines alone^13,14,16^. Given that human HSCs (Lin−CD34+CD38-CD45RA−CD90+CD49f+ long-term-(LT)-SRCs), if their stemness is maintained, are expected to increase 3–5 fold in 5–7-day cultures (estimated median doubling-time 36–48 hours), that LT-SRC display delayed G_0_ exit (1st division ~66–75 h), that short-term-SRC proliferate more rapidly, and that HSC develop in micro-environments providing additional regulatory cues^20–22^, we and others have hypothesised that chromatin-modifying agents not only expand the CD90+HSC subset without differentiation and by symmetrical division^19^, but also convert more mature CD90− HSPCs back to CD90+HSPCs.

To test this hypothesis, we cultured overnight cytokine primed human umbilical cord blood (UCB) CD133+ HSPCs on nanofibre scaffolds in serum-free media containing SCF, FL and TPO^23,24^ plus either the HDACi Scriptaid or vehicle control and examined Lin−CD34+CD38−CD45RA−CD90+CD49f+ HSPC yield. Here, we show that CD90 was upregulated on CD90− HSPCs after Scriptaid-treatment and ‘stemness’ genes were maintained in the purified CD90+ subset. Transcriptomic signatures using RNAseq and single cell q-RT-PCR of the sorted Lin−CD34+CD38−CD45RA−CD90+CD49f+ HSPC fraction following Scriptaid-treatment thus support the view that this chromatin-modifying agent can maintain more primitive HSPCs without compromising their phenotypic and transcriptomic stem cell characteristics, both by direct effects on HSC and possibly also converting CD90− more mature HSPCs to less mature CD90+ cells.

## Results and Discussion

### Scriptaid increases LTC-IC during short-term *ex vivo* culture

**Figure 1:**
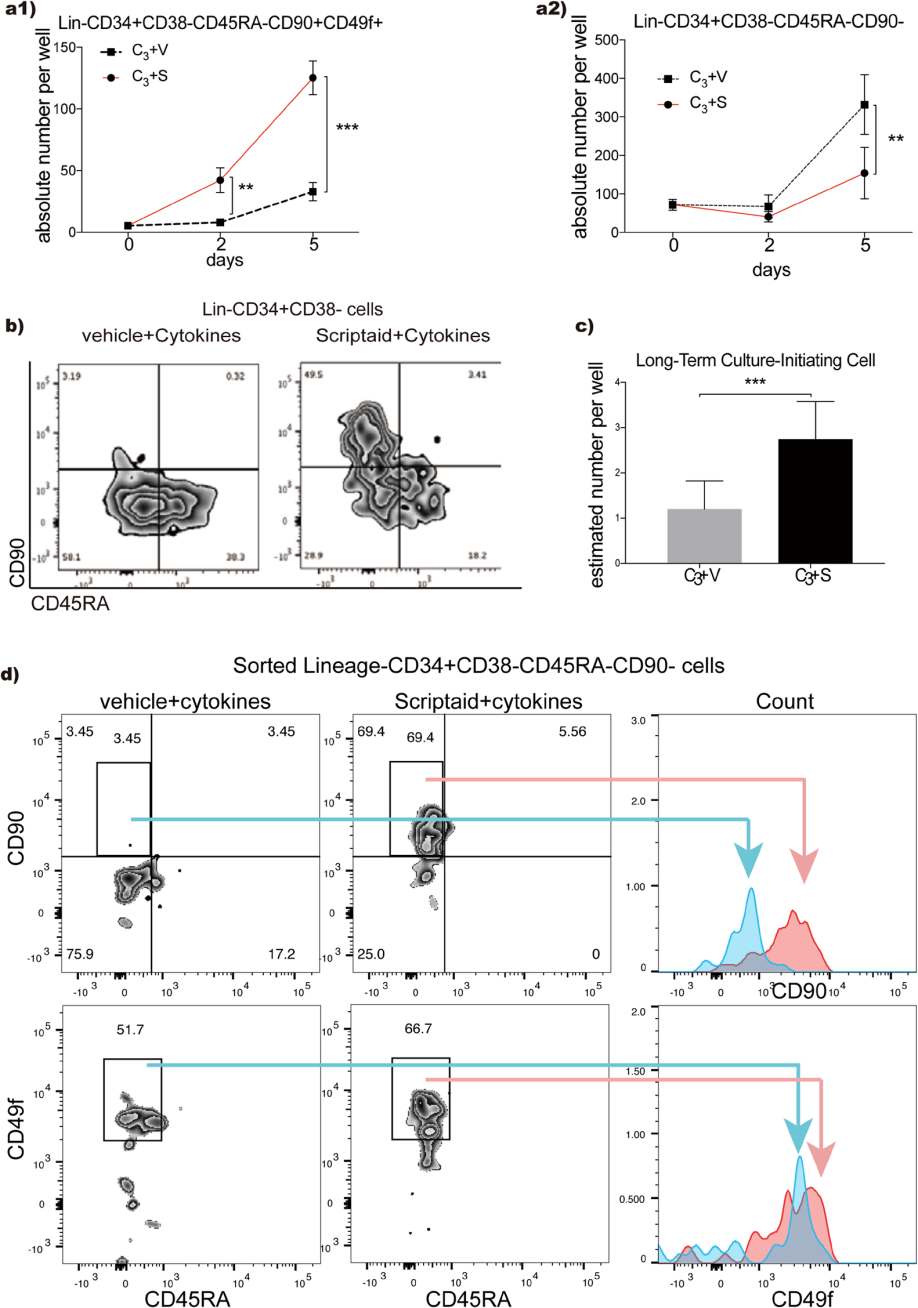
Haematopoietic stem/progenitor cell expansion *ex vivo*. (**a1**) Absolute numbers of phenotypically-defined Lin−CD34+CD38−CD45RA−CD90+CD49f+ HSPCs increased significantly with C_3_-cytokine plus Scriptaid compared to the vehicle control (**p < 0.005; ***p < 0.001. n = 6–9). (**a2**) Lin−CD34+CD38−CD45RA−CD90− cells decreased in C_3_-cytokines plus Scriptaid compared to vehicle cultures (**p < 0.005. n = 6–9). (**b**) Flow plot of 5-day expanded UCB CD133+ cells in vehicle conditions and Scriptaid conditions (right) on the expression of CD45RA against CD90. (**c**) Estimated number of LTC-IC per well in 5 day Scriptaid vs vehicle control expansion cultures based on LDA results. (n = 6–9, ***p < 0.001). (**d**) shows CD90− cells becoming CD90+ in the presence of Scriptaid. Cells negative for CD90 (Lin−CD34+CD38−CD45RA−) were sorted and then cultured in C_3_-cytokine-containing medium supplemented with Scriptaid. Representative flow cytometric analyses are shown. Multiple experiments (n = 3) demonstrate that on average 72 ± 3.1% of MPPs were CD90 positive by two days of culture (***p < 0.001), while CD49f expression levels were only slightly increased (N.S. p = 0.05) on these cultured MPPs. See Supplementary Fig. S2d,e for Median Fluorescence intensity values.

After ovenight recovery in the same 3 cytokines, cryopreserved CD133+ UCB HSPCs were cultured on nanofibre scaffolds with 3 cytokines (C_3_; SCF{stem cell factor}, TPO {thrombopoietin} and FL {Flt-3 ligand})^23,24^ plus Scriptaid^12^. Our previous studies have shown that these nanofibre scaffolds enhance human UCB CD34+/CD133+ HSPC expansion by more than 2–3 fold over a shorter period of culture than in their absence when using the same serum-free media and cytokines^23,24^. Scriptaid increased the number of Lin−CD34+CD38−CD45RA−CD90+CD49f+ HSPCs compared to cytokines alone within 2 days, and for at least 5 days, of culture (Fig. 1a1,b; *p* < *0.005* and <*0.001* respectively; Supplementary Fig. S1). To confirm this increase, Lin−CD34+CD38−CD45RA−CD90+CD49f+ cells were sorted from day-5 Scriptaid-treated or vehicle-treated cytokine-expanded cultures into long term culture-initiating cell (LTC-IC) assays, an accepted *in vitro* clonogenic assay for more immature haematopoietc progenitors^25^. This showed an absolute increase in LTC-IC/well for Scriptaid-treated cells (Fig. 1c, *p* < *0.001*) supporting the contention that Scriptaid promotes the expansion of more immature HSPCs *in vitro* (Fig. 1c). This increase in Lin−CD38−CD34+CD45RA−CD90+CD49f+ cells was accompanied by a concomitant decrease in Lin−CD38−CD34+CD45RA−CD90− MPPs (Mulipotent progenitors; Fig. 1a2,b; *p* < *0.005;* Supplementary Fig. S1). Scriptaid also restricted the cytokine-driven increase in absolute numbers of lineage-positive cells (*p* < *0.05*), LMPP (lymphoid primed multipotent progenitors; *p* < *0.005*) and GMP (granulocyte-macrophage progenitors; *p* < *0.005*), despite no increase in non-viable/apoptotic cells (Supplementary Fig. S2a–c).

When added *in vitro*, HDACi are hypothesised to transiently reactivate HSC symmetrical cell division and/or prevent HSC differentiation^9,11,12,16,18,19^. The increase we observed in Lin−CD34+CD38−CD45RA−CD90+CD49f+ cells in Scriptaid and C_3_-cytokines was considerably higher than expected for HSC symmetrical division over this short time-period, suggesting CD90+ cell generation from the CD90− HSPC subset. To determine if Scriptaid directly converts CD90− to CD90+ HSPCs, sorted MPPs (Lin−CD34+CD38−CD45RA−CD90−) were cultured with C_3_-cytokines plus Scriptaid or vehicle control. Within 48-hours, most uncultured CD90− MPPs became CD90+ in Scriptaid-treated cytokine-expanded cultures (Figs 1d and S2d,e; *p* < *0.001*). Within the Lin−CD34+CD38−CD45RA−CD49f+ population, CD90+ cell subsets are reported to contain 2–3 fold more HSCs than the CD90− subset^26^. LDA analysis also indicated higher LTC-IC frequencies in unexpanded CD90+ cells (1:12.9) than unexpanded CD90− cells (1 in 63.5; n > 4, p < 0.01; Supplementary Fig. S2f).

### RNA-sequencing reveals relative transcriptomic homogeneity of Lin−CD34+ CD38−CD45RA−CD90+ CD49f+ HSPCs after Scriptaid treatment

**Figure 2:**
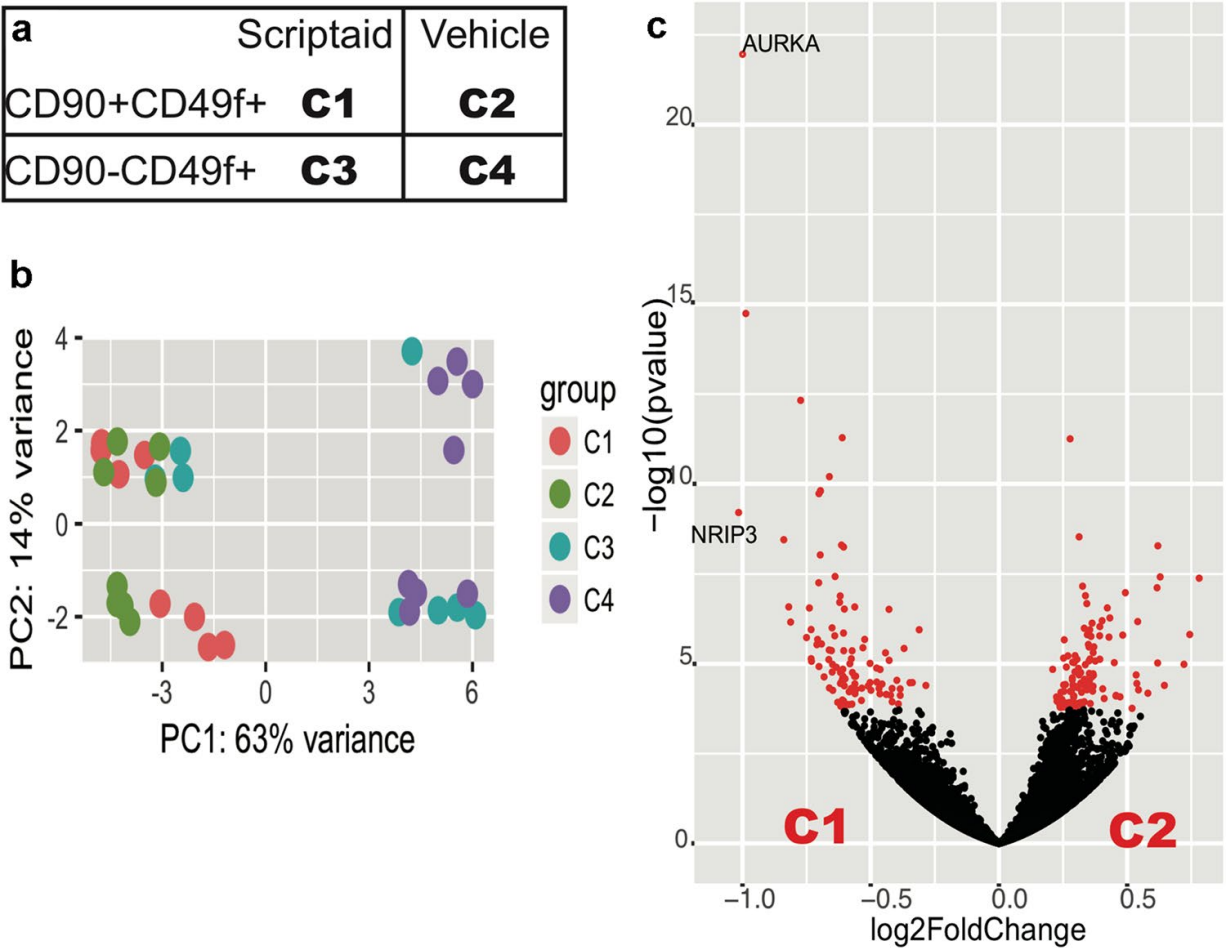
RNA sequencing of sorted CD90− and CD90+ cells after culture in C_3_-cytokines with Scriptaid or vehicle control. (**a**) RNA sequencing was performed on CD133+ UCB cells which had been expanded for 5-days in culture with C_3_-cytokines plus Scriptaid or vehicle control and then flow-sorted into: Lin−CD34+CD38−CD45RA−CD90+CD49f+ (CD90+CD49f+) cells (C1: Scriptaid-treated cultures), Lin−CD34+CD38−CD45RA−CD90+CD49f+ (CD90+CD49f+) cells (C2: Vehicle-treated cultures), Lin−CD34+CD38−CD45RA−CD90−CD49f+ (CD90−CD49f+) cells (C3: Scriptaid-treated cultures) and Lin−CD34+CD38−CD45RA−CD90−CD49f+ (CD90−CD49f+) cells (C4: Vehicle treated cultures) n = 8 for each group. (**b**) Unsupervised Principal component analysis of all 32 samples showing the clustering of C1 with C2 and three replicates of C3, but not C4. (**c**) Volcano plot shows that majority of the genes in C1 or C2 (right) populations are not significantly different (absolute fold change < 1.5).

Although HDACi appear to reprogram UCB CD34+ cells by upregulating *OCT4*, *SOX2* and *NANOG* pluripotency genes^12^, it is unclear if the CD90 upregulation we observe is associated with expression of other ‘stemness’ genes. To better understand Scriptaid’s mode of action, global gene expression profiles were compared using RNA-sequencing of bulk, flow-sorted Lin−CD34+CD38−CD45RA−CD90+CD49f+ (C1, C2) or Lin−CD34+CD38−CD45RA−CD90−CD49f+ (C3, C4) cells^26,27^ after 5-day UCB CD133+ cell-culture with C_3_-cytokines plus Scriptaid or vehicle control (Figs 2a and S3a–d). Principal component analyses (PCA, Fig. 2b) show that the Lin−CD34+CD38−CD45RA−CD90−CD49f+ cells purified after Scriptaid-treatment (C1) cluster closely with the same population in vehicle-treated cultures (C2). Only 220 genes (all fold-change < 1.5) were differentially-expressed between Lin−CD34+CD38−CD45RA−CD90+CD49f+ cells purified from Scriptaid-treated (C1) compared to vehicle-treated cultures (C2) (Fig. 2c; Supplementary Fig. S3e) consistent with their relative homogeneity.

**Figure 3:**
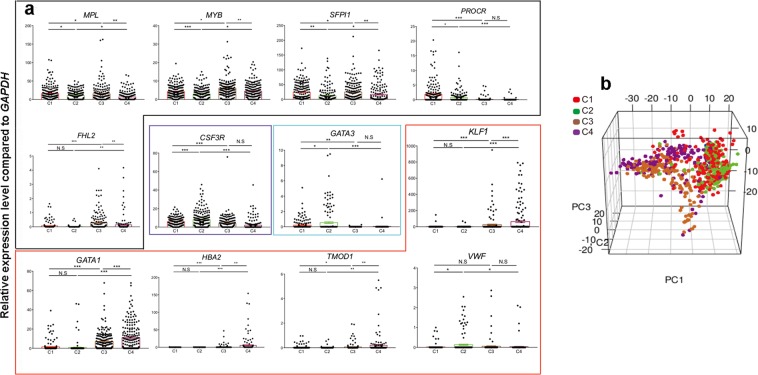
BioMark single cell q-RT-PCR of sorted CD90− and CD90+ cells after vehicle control or Scriptaid expansion. (**a**) Individually plotted relative gene expression levels of single cells (black outline: HSC-associated genes; purple outline: Myeloid-associated genes; blue outline: Lymphoid- and HSC-associated genes and red outline: Erythroid or platelet-associated genes from day 5 Scriptaid-expanded Lin−CD34+CD38−CD45RA−CD90+CD49f+ cells (C1), vehicle-expanded Lin−CD34+CD38−CD45RA−CD90+CD49f+ cells (C2), Scriptaid-expanded Lin−CD34+CD38−CD45RA−CD90−CD49f+ cells (C3), vehicle-expanded Lin−CD34+CD38−CD45RA−CD90−CD49f+ cells (C4). *P* values were generated using one-way ANOVA with multiple comparisons on Prism 7.0 (*p < 0.05; **p < 0.005; ***p < 0.001). (**b**) Three-dimensional Principal Component Analysis (PCA) with the variance between individual cells for 34 reference genes and 50 selected differentially expressed (DE) genes plotted. CD90−CD49f+ (C3 and C4) cells can be clearly distinguished from CD90+CD49f+ (C1 and C2) cells from both day 5 vehicle and Scriptaid expanded cell cultures.

To confirm the maintenance of a stemness signature by Scriptaid, single cells from C1–C4 expanded fractions were sorted, and expression of stem cell/lineage-affiliated reference genes and the top differentially-expressed genes in CD90+ versus CD90− vehicle-treated cultures (Supplementary Fig. S3h,l) were measured by qRT-PCR (Supplementary Table S1; Fig. 3a; Supplementary Figs S3i–k and S4). PCA showed close clustering of the sorted C1 and C2 populations (Fig. 3b), confirming RNA-sequencing data and consistent with LTC-IC evidence of ‘stemness’ preservation. The sorted C1 and C2 subsets were enriched to similar levels for such HSC-associated genes as *GFI1, HMGA2, EMCN* and *MEIS1*, compared to C3 and C4 subsets. Although Scriptaid had modest effects overall on the transcriptome (comparing C1 and C2), single cell q-RT-PCR revealed significantly increased expression of several HSC-associated genes in the sorted C1 versus C2 cells (*PROCR*, *MPL*, *MYB* and *SFPI1)* and reduced expression of *HLF, CSF3R, GATA3* and *VWF* suggesting Scriptaid may promote expansion of HSCs while maintaining them in a primitive state (Fig. 3a). In addition to the previously reported HSC marker PROCR^28,29^, we identified 12 genes (*CDCP1, EMCN, PDE1A, HMGA2, TMEM200A, TNFSF8, SLC7A8, EPB4IL3, GPB5, HLF, GATA3*, *SKAP1)* significantly enriched in sorted Lin−CD34+CD38-CD45RA−CD90+CD49f+ subsets (C1/2) compared to the sorted Lin−CD34+CD38−CD45RA−CD90−CD49f+ subsets (C3/4).

Recently, Velten *et al*.^30^. proposed a cellular Continuum of Low-primed UnDifferentiated (‘’CLOUD”) HSPCs containing phenotypic MPPs and MLPs, which rather than representing discrete progenitor cells, are transitory states. They identified co-expressed gene-modules associated with lineage-direction/degree of priming where the least primed-state expressed the *HOXA3/HOXB6* gene-module, while immature HSCs highly expressed an *HLF/ZFP36L2* gene-module. Notably, our detection of *HOXA3/HOXB6* (*SLC7A8, SKAP1*) and *HLF/ZFP36L2* module-genes (*HLF, EMCN, CDCP1*, *GATA3*, *MFAP2, GPAT3, OAS1, OAS3*, *UCHL1*) suggests that the Scriptaid- and vehicle- expanded and sorted C1/C2 cells still contain heterogeneous populations of immature HSCs and HSCs primed to enter all cell lineages. Downregulation of *HLF* and *GATA3* and upregulation of *SLC7A8* and *PROCR* in sorted C1 cells after Scriptaid-treatment suggests that Scriptaid may be directing HSCs and Lin−CD34+CD38−CD45RA−CD90−CD49f+ MPPs towards the least primed-state in the HSC hierarchy, while enhancing their absolute numbers. Of further note, *GATA1/KLF1* erythroid-primed-module genes^25^ were increased in the sorted C3/C4 compared to sorted C1/2 cells (Fig. 3a; Supplementary Fig. S4). Interestingly, the majority of these are significantly lower in the sorted C1/2 and C3 (Scriptaid-treated) subsets compared to the sorted C4 (vehicle-treated) subset. As several stem cell-associated genes (*MPL, MYB, FHL2*) were increased in C3 cells, Scriptaid may direct priming of Lin−CD34+CD38−CD45RA−CD90−CD49f+ MPPs away from the megakaryocyte-erythroid lineage to a more immature state.

Combining cytokines (including SCF and TPO) with Class I/II HDACis (such as Scriptaid or VPA^12^) for HSC expansion may, at first sight, appear to be counterintuitive, given that SCF and TPO have been shown to stabilise HIF-1α which has been reported to positively influence mammalian HSC self-renewal^31–34^, while Class I/II HDACis are known to repress HIF-1α function^31,35^. However, controversy surrounds the importance of HIF-1α in haematopoiesis, with Vukovic *et al*.^36,37^. more recently demonstrating that HIF-1α is not essential for ‘young’ HSC self-renewal, long term *in vivo* haematological reconstitution nor sustaining haematopoiesis post-injury, at least in mice. In their studies, Hoffman’s group^12,18^ also primed human UCB CD34+ cells with cytokines (SCF, TPO, FL, IL-3) for 16 hours, but then compared the effects of adding VPA with or without added cytokines for 4–7 days *ex vivo*. By combining these cytokines with VPA, engraftable HSC numbers were significantly enhanced. Since HSC stemness is characterised by condensed, immature mitochondria, low metabolic states and high glycolytic activity^31,38,39^, they then examined if these characteristics are maintained *ex vivo* in HSC in the presence of VPA^18^. They showed that addition of VPA to their cytokine based cultures allowed the HSCs, a least in the short term, to retain or acquire a mitochondrial profile similar to uncultured HSCs and to show enhanced glycolytic potential^18^. They further reported that the upregulation of p53 and activation of the p53-MnSOD axis were key to limiting reactive oxygen species (ROS) levels, thereby promoting HSC self-renewal and engraftable HSC expansion^18^. It remains unclear if cytokines such as SCF, which reduce ROS levels in HSCs^38,39^, contribute to these HSC effects in a manner not involving HIF-1α. Given these results, it seems likely that Scriptaid would promote immature HSPC expansion in a manner similar to VPA.

## Conclusion

In conclusion, the rarity of human haematopoietic stem cells and the very limited ability to study these cells *in vivo* in the human stem cell niche are significant hurdles in understanding the regulatory networks and cues that control the balance between their maintenance, survival, quiescence, self-renewal and differentiation, and may limit their clinical use. Our studies support the single-cell view that specific HDACi, when combined with cytokines, maintain the transcriptome of more immature HSPCs. This has the potential to impact on outcomes of *ex vivo* gene editing for hematological diseases, where short-term *ex vivo* cultures require HSC maintenance or expansion.

## Methods

### Cell isolation

Human umbilical cord blood (UCB) was collected from the John Radcliffe Hospital, Oxford, UK or provided via the NHS Cord Blood Bank, London, and used with informed, written pre-consent and ethical approval from the South Central Oxford C and Berkshire Ethical Committees and approval of the NHSBT R&D committee and all methods were performed in accordance with the relevant guidelines and regulations. Mononuclear cells (MNCs; density < 1.077 g/ml) were isolated by density gradient centrifugation no more than 24 hours after UCB collection. Human CD133+ haematopoietic stem and progenitor cells (HSPC) were enriched by MACS using the CD133 direct microbead kits (Miltenyi Biotec GmbH) and cryopreserved until use^23,24,40,41^. Purity of the cells isolated was routinely assessed by flow cytometry and only donors with >90% CD133+ cell purity were used for expansion experiments.

### Expansion culture

Cells were thawed and cultured overnight (20–24 hs) in 24 or 96 well round bottom plates at 200 cells/μl in serum free Stem Span ACF media (Stem Cell Technologies) supplemented with 3 cytokines (C3) at 37 °C, 5% CO_2_, 95% humidity. The following cytokines were used for expansion: Stem Cell Factor (SCF), FLT-3Ligand (FL) (both at 100 ng/ml) and Thrombopoietin (TPO; 20 ng/ml) (all from R&D Systems). On day 0, cells were harvested, counted using Countbright absolute counting beads (Molecular Probes) by flow cytometry^40–42^ and plated on 3D nanofiber scaffolds (NANEX plates from Compass Biomedicals) at an optimised cell density of 2,500 cells/ml in Stem Span ACF supplemented with 3 cytokines as described above^23,24,41^. Cells were either treated with the HDAC inhibitor Scriptaid (Sigma) at 1 µM in DMSO or an equivalent amount of vehicle (0.1% DMSO, Sigma) and harvested for downstream assays on days 2 or 5.

### Flow cytometry

Multicolour flow cytometry characterization of CD133^+^ human UCB-derived HSPC was performed according to Notta *et al*.^26,41^. The following antibodies (Supplementary Table S2) against human cell surface antigens were used: CD34 AF700 (581) or APC (8G12), CD133 PE or APC (293C3), CD45RA APC-H7 or FITC (HI100), CD38 PE-TxR or BB515 (HIT2), CD90 PE or PE-Cy7 (5E10), CD123 PerCP-Cy5.5 or PE-Cy7 (6H6), CD49f PE-Cy7 or PerCP-Cy5.5 (GoH3), CD184 APC (12G5), and a panel of lineage markers conjugated to PE-Cy5: CD2 (RPA-2.10), CD3 (HIT3a), CD4 (RPA-T4), CD8a (RPA-T8), CD7 (6B7), CD10 (HI10a), CD11b (ICRF44), CD14 (61D3), CD19 (HIB19), CD20 (2H7), CD235ab (HIR2) and CD56 (B159). Briefly, cells were resuspended in human FcR blocking reagent diluted in MACS buffer (both from Miltenyi Biotec GmbH) and incubated for 10 min. at 4 °C. The cells were then incubated with a mixture of fluorescently labelled antibodies diluted in MACS buffer for 20 min on ice. Cells were washed once, resuspended in MACS buffer and acquired immediately on an LSRII flow cytometer (BD Biosciences). DAPI (Invitrogen) was added at 100 ng/ml directly before acquisition to distinguish live and dead cells. Data were analysed with FlowJo software (TreeStar Inc.). Cell subsets were determined by gating on single viable, Lin^−^ (lineage negative) cells, and defined as HSC (haematopoietic stem cells): Lin−CD38−CD34+CD45RA−CD90+CD49f+ ; MPP (multipotent progenitors): Lin−CD38−CD34+CD45RA−CD90−; LMPP (lymphoid primed multipotent progenitors): Lin−CD38-CD34+CD45RA+CD90−; CMP (common myeloid progenitors): Lin−CD38+CD34+CD45RA−CD123+; GMP (granulocyte-macrophage progenitors): Lin−CD38+CD34+CD45RA+CD123+; and MEP (megarkaryocyte-erythroid progenitors): Lin−CD38+CD34+CD45RA−CD123− and HSPC (haematopoietic stem and progenitor cells): Lin−CD34+CD133+ ^26,32^. A representative gating strategy is shown in Supplementary Fig. S5. In some cases, sorting of these HSC and progenitor subpopulations was carried out on a FACS Aria II (BD Biosciences)^40^.

### Apoptosis assay

All expanded cells were harvested after 5 days of culture and stained with Annexin V FITC (BD Biosciences) according to the manufacturer’s instructions. DAPI was added at 100 ng/ml directly before acquisition on a LSRII flow cytometer (BD Biosciences). Data were analysed with FlowJo software (TreeStar Inc.). Cells were gated on FSC-A against SSC-A and then analysed for apoptosis (AnnexinV+/DAPI−) and cell death (AnnexinV+/DAPI+).

### LTC-IC and CFU assays

Long-term culture-initiating cells (LTC-ICs) assay were following the protocol described previously^25^. The murine stromal cell lines (M2-10B4 and SL/SL mixed at 1:1) and irradiated with 8000 cGy were plated in 96-well collagen-coated microtiter plates (5000 cells/well of each cell line) and cultured in long-term culture medium (MyeloCult H5100; Stem Cell Technologies) supplemented with hydrocortisone 21–hemi-succinate (10^−6^ M). Limiting dilution analysis (LDA) for quantitating LTC-ICs present in the Lin−CD34+CD38−CD45RA−CD90+CD49f+ cell subset 5 days after expansion in C_3_-cytokines plus Scriptaid or the vehicle control. Cells were plated at 50 to 150 cells per well (10–100 for unexpanded condition) by flow sorting^43^. Co-cultures were maintained at 37 °C in high humidity and with 50% medium exchange every week. After 6 weeks, all cells were plated in methylcellulose cultures supplemented with complete methylcellulose-based medium (MyeloCult H4435; Stem Cell Technologies)^25,42,43^. Long-term culture colony-forming cells (LTC-CFCs; readout of LTC-IC assay) were scored after an additional 14–16 days of culture^25,43^. Limiting Dilution Assay (LDA) analysis was performed on ELDA (http://bioinf.wehi.edu.au/software/elda/) based on the number of negative wells (showing no CFCs) in each condition with a confidence interval of 0.95.

### High-throughput RNA sequencing and bioinformatics analysis

Expanded cells with the phenotype of Lin−CD34+CD38−CD45RA−CD49f+ were index sorted (BD FACS Aria II cell sorter) into 4 µl lysis buffer as 100 cells per sample based on the expression of CD90. The mRNA of quadruplicate samples originating from two biological replicates were then used to prepare the cDNA library following the protocol described by Picelli and colleagues^44^. Amplified cDNA libraries were cleaned by Ampure SPRI beads (1:1) and then quality checked using the Agilent high-sensitive analysis chip. About 1 ng of each library was processed for library preparation using the Nextera XT DNA sample preparation kit (Illumina) following the manufacturer’s instructions. After cleaning with Ampure beads, samples were pooled to a final concentration of 10 nM. The pooled library was sequenced in multiple lanes using the Illumina HiSeq4000 platform (pair-end 75-bp reads) at the Wellcome Trust Centre for Human Genetics, University of Oxford, UK. Approximately thirty (30) million reads per sample were obtained. Raw files of the same sample from different lanes were merged into a single file using Samtools (1.3). PCR duplications were checked and removed by the MarkDuplicates function on Picardtools (2.3.0) and Samtools. Processed files were then aligned using STAR (2.4.2a) against the human genome (Homo_sapiens/Ensembl/GRCh37) and the quality was also checked by fastQC. Genes were annotated using BioMart (2.32.1) and non-adjusted read counts for each gene were assessed statistically for global differential expression between the specified populations using the RUVseq (1.10.0) and DESeq2 (1.16.1) packages on R (3.4.3). Genes that are significant (absolute fold change >1.5 or <0.5) at a 5% false discovery rate (calculated using a Benajmini-Hochberg adjusted p-value) are considered differentially expressed between populations as described^45^. Gene Ontology Biological Process analysis was performed on R by package topGO (3.6).

### TaqMan gene expression analysis

Multiplex quantitative real time PCR (q-RT-PCR) analysis was performed with the BioMark 96.96 Dynamic Array platform (Fluidigm) and TaqMan Gene Expression Assays (Applied Biosystems) as previously described^46^. Inventoried or Made-to order TaqMan assays (Supplementary Table S1, Applied Biosystems) were pooled to a final concentration of 2x for each assay. Individual cells were sorted directly into RT-PreAmp Master Mix (2.5 µl Reaction Mix (Invitrogen); 1.25 µl 2x assay pool; 0.6 µl RT/Taq enzyme (Superscript III kit, Invitrogen). Cell lysis and sequence specific reverse transcription were performed at 50 °C for 15 min. The reverse transcriptase was inactivated by heating to 95 °C for 2 min. Subsequently, in the same tube, cDNA was pre-amplified by denaturing at 95 °C for 15 s, and annealing and amplification at 60 °C for 4 min for 22 cycles. PCR products were diluted 5-fold prior to analysis with Universal PCR Master Mix and inventoried TaqMan gene expression assays (ABI) in 96.96 Dynamic Arrays on the BioMark Fluidigm System. Ct values were calculated from the system’s software (BioMark Real-time PCR Analysis; Fluidigm). Data were analysed using the ΔΔCt method and results were normalized to *GAPDH* expression. Only cells expressing *GAPDH* were included in the analysis. Principal component analysis was performed using the fluidigmSC package (3.6.2) in R (3.4.3) using LoD as 24 on raw Ct value.

## Supplementary information

Supplementary Information

## Data Availability

The datasets generated during and/or analysed during the current study are available from the first author on reasonable request.
