# Supplementary material for: Single-cell assessment of transcriptome alterations induced by Scriptaid in early differentiated human haematopoietic progenitors during ex vivo expansion
Source: Sci Rep. 2019 Mar 28;9:5300. doi: 10.1038/s41598-019-41803-z (PMC6438964; doi:10.1038/s41598-019-41803-z)

Supplementary Information

Single-cell assessment of transcriptome alterations induced by Scriptaid in early differentiated human haematopoietic progenitors during *ex vivo* expansion

Peng Hua[1,2], Barbara Kronsteiner[2], Mark van der Garde[2], Neil Ashley[1], Diana Hernandez[3], Marina Tarunina[3], Lilian Hook[3], Yen Choo[3], Irene Roberts[1,4,5], Adam Mead[1,5] and Suzanne M. Watt[2].

[1]MRC Molecular Haematology Unit, Weatherall Institute of Molecular Medicine, Radcliffe Department of Medicine, University of Oxford, John Radcliffe Hospital, Oxford, OX3 9DS, UK; [2]Stem Cell Research, Nuffield Division of Clinical Laboratory Sciences, Radcliffe Department of Medicine, University of Oxford, and NHS Blood and Transplant, John Radcliffe Hospital, Oxford, OX3 9BQ, UK; [3]Plasticell Ltd, Stevenage Bioscience Catalyst, Stevenage, SG1 2FX, UK; [4]Department of Paediatrics, University of Oxford, Children's Hospital, John Radcliffe Hospital, Oxford OX3 9DU, UK; [5]Haematology Theme, Oxford Biomedical Research Centre, Oxford University Hospitals, Oxford, UK.

Statement of equal author contribution: P.H. and B.K. contributed equally to this study.

Supplementary Tables

Supplementary Table S1. Taqman probes used for Biomark Single-cell qPCR:

Reference genes	Annotation	TAQMAN id
BMI1 (HSC)	BMI1 Proto-Oncogene, Polycomb Ring Finger	Hs00995536_m1
FLT3 (HSC)	fms-related tyrosine kinase 3	Hs00975643_m1
GFI1 (HSC/homing)	Growth Factor Independent 1 Transcriptional Repressor	Hs00382207_m1
HMGA2 (HSC/fetal)	High-Mobility Group (Nonhistone Chromosomal) Protein Isoform I-C	Hs04397751_m1
ITGA6 (HSC)	Integrin Subunit Alpha 6 (CD49f)	Hs01116228_m1
JUN (HSC)	Jun Proto-Oncogene, AP-1 Transcription Factor Subunit	Hs01103582_s1
MCL1 (HSC)	Myeloid Cell Leukemia Sequence 1 (BCL2-Related)	Hs01050896_m1
MEIS1 (HSC)	Meis homeobox 1	Hs00180020_m1
MPL (HSC)	myeloproliferative leukemia virus oncogene	Hs00180489_m1
MYB (HSC)	Proto-Oncogene C-Myb	Hs00920556_m1
MYC (HSC)	V-Myc Avian Myelocytomatosis Viral Oncogene Homolog	Hs00153408_m1
MYCN (HSC)	V-Myc Avian Myelocytomatosis Viral Oncogene Neuroblastoma Derived Homolog	Hs00232074_m1
POU5F1 (HSC)	Oct-04	Hs00999634_gH
STAT5a (HSC)	Transcription Factor STAT5A	Hs00559648_g1
STAT5b (HSC)	Transcription Factor STAT5B	Hs00560026_m1
CD79A (Lymphoid)	Membrane-Bound Immunoglobulin-Associated Protein	Hs00998119_m1
EBF1 (Lymphoid)	Early B-Cell Factor 1	Hs01092694_m1
GATA3 (Lymphoid)	Trans-Acting T-Cell-Specific Transcription Factor GATA-3	Hs00231122_m1
IL3RA (Lymphoid)	Interleukin-3 Receptor Subunit Alpha	Hs00608141_m1
PAX5 (Lymphoid)	B-Cell Lineage Specific Activator	Hs00277134_m1
PTCRA (Lymphoid)	Pre T-Cell Antigen Receptor Alpha	Hs00300125_m1
RAG1 (Lymphoid)	Recombination Activating Protein 1	Hs00172121_m1
CEBPA (Myeloid)	CCAAT/Enhancer Binding Protein Alpha	Hs00269972_s1
CSF3R (Myeloid)	Colony Stimulating Factor 3 Receptor	Hs01114420_m1
MPO (Myeloid)	myeloperoxidase	Hs00165162_m1
SFPI1 (Myeloid/ HSC)	Hematopoietic Transcription Factor PU.1	Hs02786711_m1
EPOR (Erythroid)	Erythropoietin Receptor	Hs00959426_m1
GATA1 (Erythroid)	GATA Binding Protein 1 (Globin Transcription Factor 1)	Hs01085823_m1
KLF1 (Erythroid)	Erythroid-Specific Transcription Factor EKLF	Hs00610592_m1
VWF (Platelet)	Coagulation Factor VIII VWF	Hs01109457_m1
CXCR4 (homing/retention)	chemokine (C-X-C motif) receptor 4	Hs00976734_m1
GAS2 (Cell growth)	growth arrest-specific 2	Hs00169477_m1
ASF1A (epigenetic)	ASF1 Anti-Silencing Function 1 Homolog A	Hs00204044_m1
CHD7 (epigenetic)	Chromodomain Helicase DNA Binding Protein 7	Hs00215010_m1
GAPDH (House-keeping)	glyceraldehyde-3-phosphate dehydrogenase	Hs02758991_g1
**Genes upregulated in CD90+ cells**	**Annotation**	**TAQMAN id**
C10orf128	chromosome 10 open reading frame 128 [Source:HGNC Symbol;Acc:HGNC:27274]	Hs01393050_m1
CD22	CD22 molecule [Source:HGNC Symbol;Acc:HGNC:1643]	Hs00998488_m1
CDCP1	CUB domain containing protein 1 [Source:HGNC Symbol;Acc:HGNC:24357]	Hs01080405_m1
CRHBP	corticotropin releasing hormone binding protein [Source:HGNC Symbol;Acc:HGNC:2356]	Hs00181810_m1
EMCN	endomucin [Source:HGNC Symbol;Acc:HGNC:16041]	Hs01038204_m1

EPB41L3	erythrocyte membrane protein band 4.1 like 3 [Source:HGNC Symbol;Acc:HGNC:3380]	Hs00202360_m1
GBP5	guanylate binding protein 5 [Source:HGNC Symbol;Acc:HGNC:19895]	Hs00369472_m1
GPAT3	glycerol-3-phosphate acyltransferase 3 [Source:HGNC Symbol;Acc:HGNC:28157]	Hs00262010_m1
HLF	HLF, PAR bZIP transcription factor [Source:HGNC Symbol;Acc:HGNC:4977]	Hs00171406_m1
KIAA0087	KIAA0087 [Source:HGNC Symbol;Acc:HGNC:22191]	Hs00207421_m1
MFAP2	microfibrillar associated protein 2 [Source:HGNC Symbol;Acc:HGNC:7033]	Hs01027737_m1
OAS1	2'-5'-oligoadenylate synthetase 1 [Source:HGNC Symbol;Acc:HGNC:8086]	Hs00973635_m1
OAS3	2'-5'-oligoadenylate synthetase 3 [Source:HGNC Symbol;Acc:HGNC:8088]	Hs00196324_m1
PDE1A	phosphodiesterase 1A [Source:HGNC Symbol;Acc:HGNC:8774]	Hs00897273_m1
PROCR	protein C receptor [Source:HGNC Symbol;Acc:HGNC:9452]	Hs00197387_m1
RAI14	retinoic acid induced 14 [Source:HGNC Symbol;Acc:HGNC:14873]	Hs00210238_m1
SKAP1	src kinase associated phosphoprotein 1 [Source:HGNC Symbol;Acc:HGNC:15605]	Hs00175372_m1
SLC16A2	solute carrier family 16 member 2 [Source:HGNC Symbol;Acc:HGNC:10923]	Hs00185140_m1
SLC7A8	solute carrier family 7 member 8 [Source:HGNC Symbol;Acc:HGNC:11066]	Hs00794796_m1
TMEM200A	transmembrane protein 200A [Source:HGNC Symbol;Acc:HGNC:21075]	Hs00287589_m1
TNFSF8	tumor necrosis factor superfamily member 8 [Source:HGNC Symbol;Acc:HGNC:11938]	Hs00174286_m1
TRIM47	tripartite motif containing 47 [Source:HGNC Symbol;Acc:HGNC:19020]	Hs00386112_m1
UCHL1	ubiquitin C-terminal hydrolase L1 [Source:HGNC Symbol;Acc:HGNC:12513]	Hs00985157_m1
**Genes upregulated in CD90- cells**	**Annotation**	**TAQMAN id**
ABCB6	ATP binding cassette subfamily B member 6 (Langereis blood group) [Source:HGNC Symbol;Acc:HGNC:47]	Hs00180568_m1
APOC1	apolipoprotein C1 [Source:HGNC Symbol;Acc:HGNC:607]	Hs00155790_m1
BCL2L11	BCL2 like 11 [Source:HGNC Symbol;Acc:HGNC:994]	Hs01076940_m1
CA1	carbonic anhydrase 1 [Source:HGNC Symbol;Acc:HGNC:1368]	Hs00266139_m1
CD84	CD84 molecule [Source:HGNC Symbol;Acc:HGNC:1704]	Hs01547121_m1
CEBPD	CCAAT/enhancer binding protein delta [Source:HGNC Symbol;Acc:HGNC:1835]	Hs00270931_s1
CITED2	Cbp/p300 interacting transactivator with Glu/Asp rich carboxy-terminal domain 2 [Source:HGNC Symbol;Acc:HGNC:1987]	Hs00366696_m1
CSF2RB	colony stimulating factor 2 receptor beta common subunit [Source:HGNC Symbol;Acc:HGNC:2436]	Hs00166144_m1
CST7	cystatin F [Source:HGNC Symbol;Acc:HGNC:2479]	Hs00175361_m1
CSTA	cystatin A [Source:HGNC Symbol;Acc:HGNC:2481]	Hs00193257_m1
DLC1	DLC1 Rho GTPase activating protein [Source:HGNC Symbol;Acc:HGNC:2897]	Hs00183436_m1
ELL2	elongation factor for RNA polymerase II 2 [Source:HGNC Symbol;Acc:HGNC:17064]	Hs00603761_g1
FAM171A1	family with sequence similarity 171 member A1 [Source:HGNC Symbol;Acc:HGNC:23522]	Hs00295934_m1
FHL2	four and a half LIM domains 2 [Source:HGNC Symbol;Acc:HGNC:3703]	Hs00991866_m1
HBA2	hemoglobin subunit alpha 2 [Source:HGNC Symbol;Acc:HGNC:4824]	Hs00361191_g1
LEF1	Lymphoid Enhancer Binding Factor 1	Hs01547250_m1
MICAL2	microtubule associated monooxygenase, calponin and LIM domain containing 2 [Source:HGNC Symbol;Acc:HGNC:24693]	Hs01121809_m1
NECTIN1	nectin cell adhesion molecule 1 [Source:HGNC Symbol;Acc:HGNC:9706]	Hs01591978_m1
PLD1	phospholipase D1 [Source:HGNC Symbol;Acc:HGNC:9067]	Hs01111342_m1
PRTN3	proteinase 3 [Source:HGNC Symbol;Acc:HGNC:9495]	Hs01553330_m1
PVT1	Pvt1 oncogene (non-protein coding) [Source:HGNC Symbol;Acc:HGNC:9709]	Hs00413039_m1
RNASE2	ribonuclease A family member 2 [Source:HGNC Symbol;Acc:HGNC:10045]	Hs00795553_s1
RYR3	ryanodine receptor 3 [Source:HGNC Symbol;Acc:HGNC:10485]	Hs00168821_m1
STXBP5	syntaxin binding protein 5 [Source:HGNC Symbol;Acc:HGNC:19665]	Hs01083307_m1
TFR2	transferrin receptor 2 [Source:HGNC Symbol;Acc:HGNC:11762]	Hs01056398_m1
TMOD1	tropomodulin 1 [Source:HGNC Symbol;Acc:HGNC:11871]	Hs00268513_m1
TSPOAP1	TSPO associated protein 1 [Source:HGNC Symbol;Acc:HGNC:16831]	Hs00895494_m1

Supplementary Table S2. Antibodies Used for Flow Cytometry

Name	Clone	Isotype	Conj.	Company	Dilution
CD34	581	mIgG1	AF700	BD	1:60
CD38	HIT2	mIgG1	PETxR/BB515	Caltag/BD	1:40
CD45RA	HI100	mIgG2bk	APC-H7	BD	1:50
CD90	5E10	IgG1	PE	Biolegend	1:50
CD49f	GoH3	rIgG2a	PE-Cy7	Biolegend	1:120
CD133	293C3	IgG2b	APC	Miltenyi	1:30
CD123	6H6		PerCP-Cy5.5	Biolegend	1:50
CD2	RPA-2.10	IgG1	PE-Cy5	Biolegend	1:150
CD3	HIT3a	IgG2a	PE-Cy5	Biolegend	1:200
CD4	RPA-T4	IgG1	PE-Cy5	Biolegend	1:150
CD7	CD7-6B7	IgG1	PE-Cy5	Biolegend	1:150
CD8a	RPA-T8	IgG1	PE-Cy5	Biolegend	1:200
CD10	HI10a	IgG1	PE-Cy5	Biolegend	1:80
CD11b	ICRF44	IgG1	PE-Cy5	Biolegend	1:80
CD14	6103	IgG2a	PE-Cy5	eBio	1:80
CD19	HIB19	IgG1	PE-Cy5	Biolegend	1:200
CD20	2H7	IgG2a	PE-Cy5	Biolegend	1:150
CD56	B159	IgG1	PE-Cy5	BD	1:20
CD235ab	HIR2	IgG2b	PE-Cy5	Biolegend	1:1200
DAPI			UV 440/40		100ng/ml

Supplementary Figure Legends

Supplementary Figure S1. Flow cytometric analyses for human UCB CD133+ cells expanded in vitro.

Representative gating strategy for flow cytometry analysis for day 5 cultured cells in Figures 1a1 and 1a2. The media contained C3-cytokine plus vehicle (top) or Scriptaid (bottom). Lin-CD34+CD38-/lowCD45RA- cells were gated as shown and then selected for CD90+ or CD90- cells. The CD90+ subset was further enriched for CD49f+ cells. All gating was determined by FMO (Fluorescence Minus One).

Supplementary Figure S2. Expansion of CD133+ HSC and progenitor populations after 5 days culture with cytokines and Scriptaid or vehicle

(a) Cryopreserved CD133+ UCB cells were thawed and cultured overnight in C3-cytokine-containing serum free medium. Cells were then plated for expansion and also analysed for the frequency of HSC and progenitors. Scriptaid or vehicle was added when plating and all cells post 5-days expansion were analysed by flow cytometry. In the presence of C_3-cytokines and Scriptaid, the absolute number of Lin+, LMPP and GMP cells decreased significantly compared to vehicle. Scriptaid had no effect on the numbers of committed myeloid progenitors (CMP, MEP) (*, $p<0.05$; **, $p<0.005$; ***, $p<0.001$, n=6-9). (b-c) Proportions of AnnexinV+DAPI- (Apoptotic) and AnnexinV+DAPI+ (Dead) cells in C_3-cytokine containing medium supplemented with Scriptaid or the vehicle control did not differ on day 5 of expansion. (d-e) Median Fluorescence Intensity values of CD90-PE and CD49f-PECy7 analysed by Student's T test against vehicle control (***, $p<0.001$; N.S, $p=0.05$. n=3). (f) Limiting dilution analysis (LDA) for quantitating LTC-ICs present in the unexpanded Lin-CD34+CD38-CD45RA-CD90+/-CD49f+ cell subsets (n=4-12).

Supplementary Figure S3. Supporting information for RNA-sequencing and single-cell qPCR

(a) QC of a cDNA library from 100 sorted cells using the Agilent high-sensitivity chip. (b) QC of library pool using the Agilent high-sensitivity chip. (c-d) QC of raw fastq file from hiseq4000. (e) Sample distance heatmap illustrates C1 and C2 cells cannot be clustered based on the phenotype. (f) Sample distance heatmap illustrates C1 and C4 cells can be clustered based on the phenotype but not biological variance. (g) Sample distance heatmap illustrates C1 and C3 cells cannot be clustered based on the phenotype. (h) Volcano plot demonstrating the top deferentially expressed genes between C2 and C4 populations. (i) Principal Component Analysis (PCA) plotting the variance between individual cells for 50 selected DE genes. CD90-CD49f+ (C3 and C4) cells can be clearly distinguished from CD90+CD49f+ (C1 and C2) cells in both day 5 vehicle and Scriptaid expanded cell culture. (j) Screen plot of PCA analysis of Fluidigm single-cell qPCR data for 50 selected DE genes plus reference genes. (k) Screen plot of PCA analysis of Fluidigm single-cell qPCR data for 50 selected DE genes. (l) GO Biological Process analysis results of the DE genes between C2 and C4 populations.

Supplementary Figure S4. Transcriptome profiling of single cells

Individually plotted relative gene expression levels of single cells (34 reference genes and 50 selected DE genes) from day 5 Scriptaid-expanded Lin-CD34+CD38-CD45RA-CD90+CD49f+ cells (C1), vehicle-expanded Lin-CD34+CD38-CD45RA-CD90+CD49f+ cells (C2), Scriptaid-expanded Lin-CD34+CD38-CD45RA-CD90-CD49f+ cells (C3), and vehicle-expanded Lin-CD34+CD38-CD45RA-CD90-CD49f+ cells (C4). P values were generated using one-way ANOVA with multiple comparisons on Prism 7.0 (*, $p<0.05$; **, $p<0.005$; ***, $p<0.001$).

Supplementary Figure S5. Flow cytometric analyses for overnight cultured CD133+ cells or C_3-cytokine-expanded human UCB CD133+ cells

Representative gating strategy for flow cytometry and sorting: HSC: Lin-CD38-CD34+CD45RA-CD90+CD49f+; HSPC: Lin-CD34+CD133+; MPP: Lin-CD38-CD34+CD45RA-CD90-; LMPP: Lin-CD38-CD34+CD45RA+CD90-; CMP: Lin-CD38+CD34+CD45RA-CD123+; GMP: Lin-CD38+CD34+CD45RA+CD123+; and MEP: Lin-CD38+CD34+CD45RA-CD123-. All gating was determined by FMO (Fluorescence Minus One).

Supplementary Fig. S1. Watt. 2018. TOP.

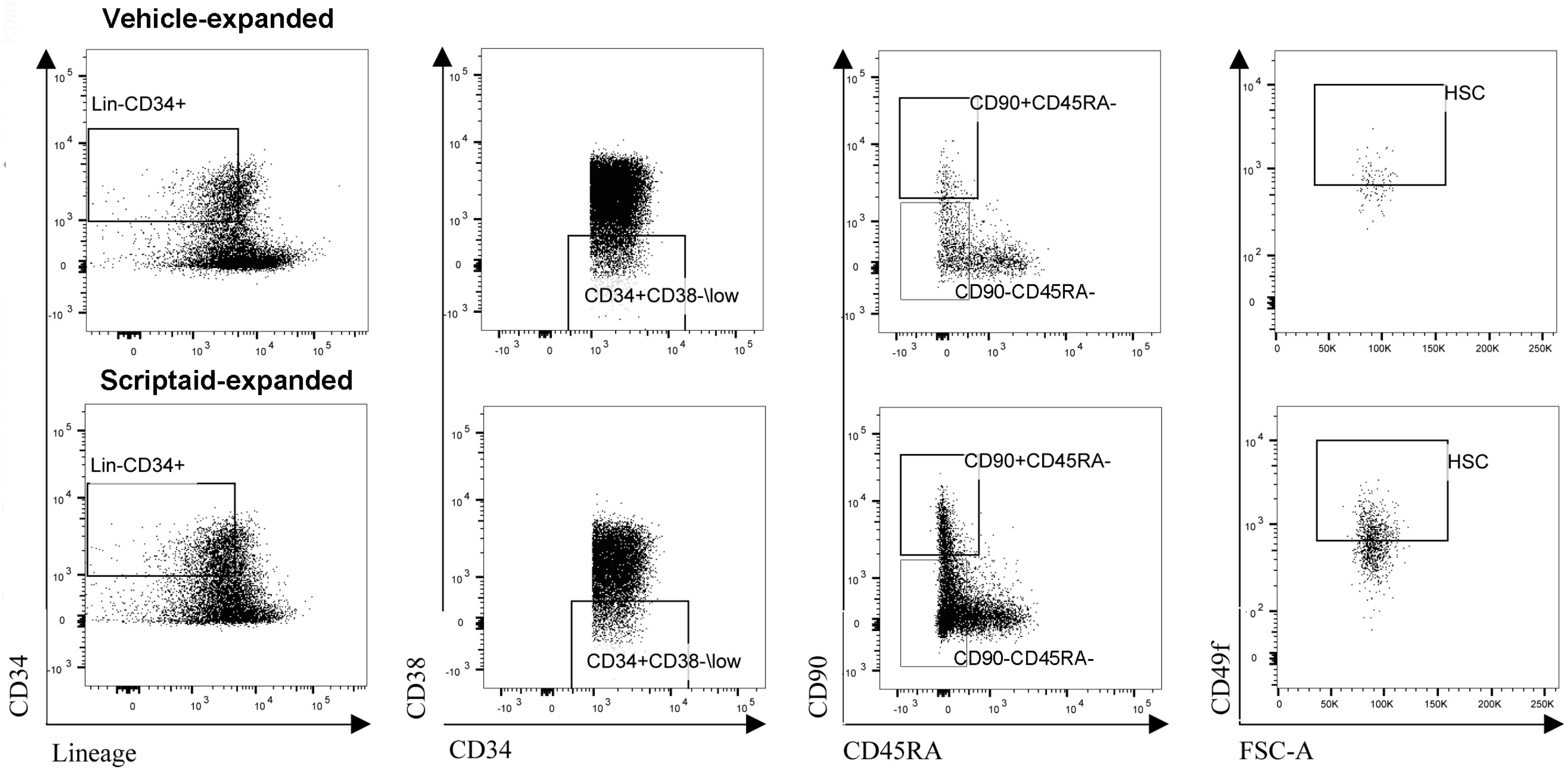

Supplementary Figure S2 Watt. 2018. TOP.

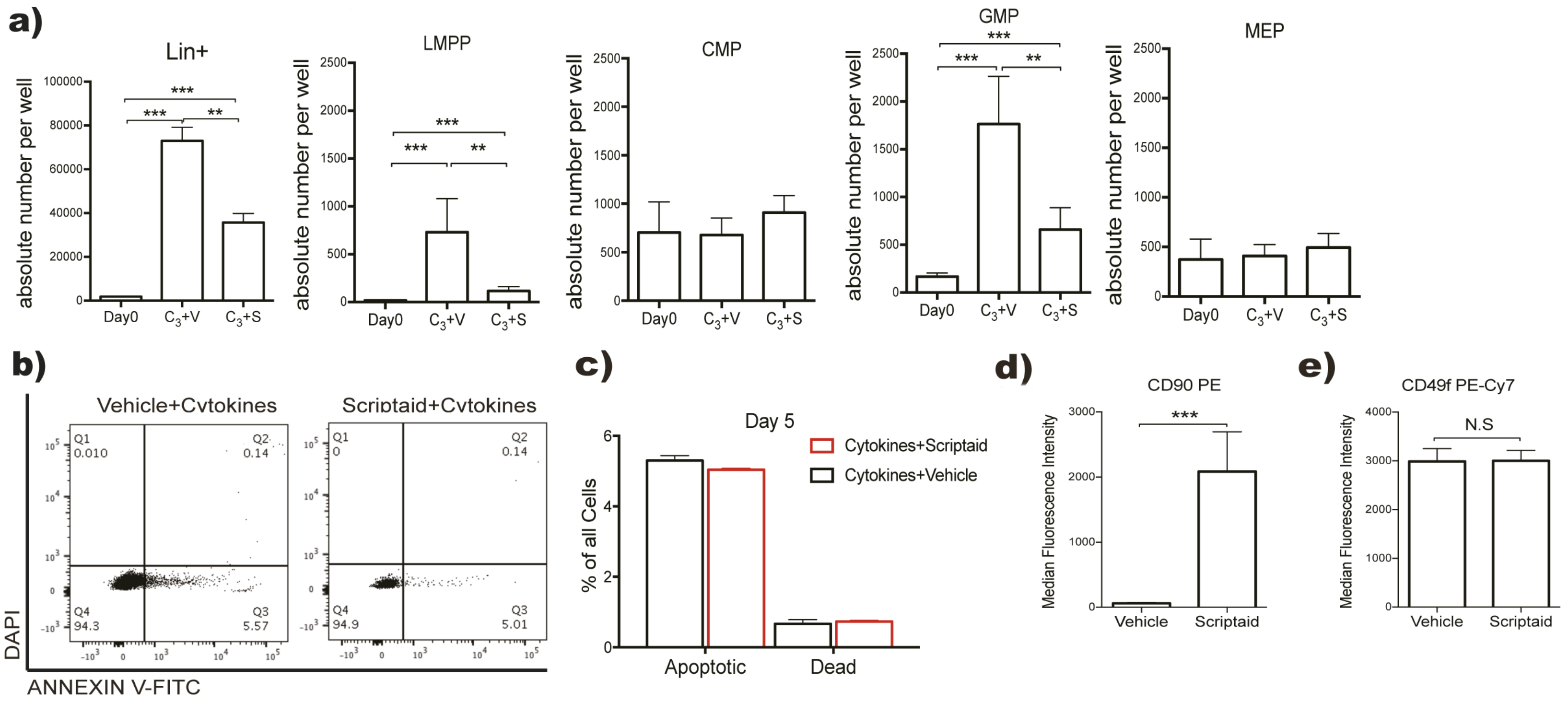

f)

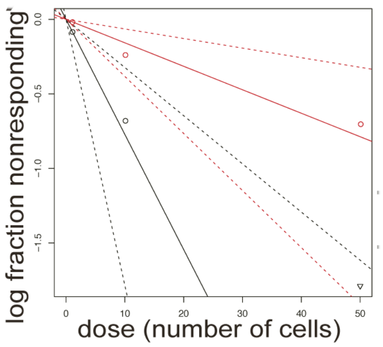

Confidence intervals for 1/(stem cell frequency)

Group	Lower	Estimate	Upper
Lin-CD34+CD38-CD45RA-CD90+CD49f+	30.9	12.9	5.6
Lin-CD34+CD38-CD45RA-CD90-CD49f+	155.4	63.5	26.1

Supplementary Figure S3 Watt. 2018. TOP.

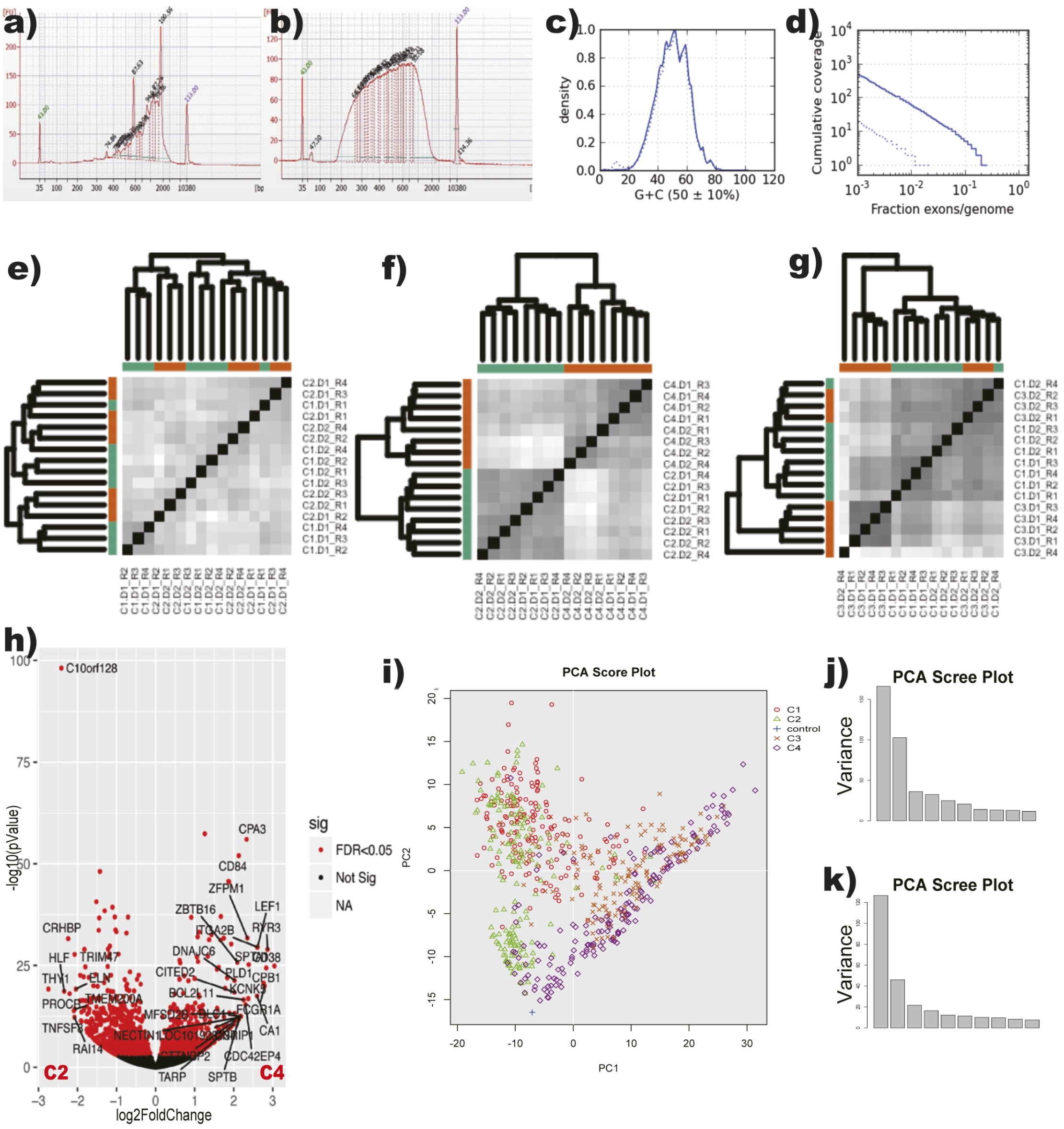

l)

	GO.ID	Term	Annotated	Significant	Expected	Rank in clas	classic	KS	weight
1	GO:1903047	mitotic cell cycle process	859	790	590.13	28	< 1e-30	< 1e-30	< 1e-30
2	GO:0006996	organelle organization	3651	3070	2508.24	4	< 1e-30	4.3e-22	< 1e-30
3	GO:0006412	translation	652	599	447.92	42	< 1e-30	3.8e-17	< 1e-30
4	GO:0016071	mRNA metabolic process	626	577	430.06	45	< 1e-30	< 1e-30	< 1e-30
5	GO:0034470	ncRNA processing	327	318	224.65	58	< 1e-30	1.6e-07	< 1e-30
6	GO:0022613	ribonucleoprotein complex biogenesis	377	357	259	71	< 1e-30	1.1e-14	< 1e-30
7	GO:1902582	single-organism intracellular transport	1715	1453	1178.21	33	< 1e-30	2.3e-10	< 1e-30
8	GO:0032446	protein modification by small protein co...	891	786	612.12	49	< 1e-30	7.4e-05	< 1e-30
9	GO:0006974	cellular response to DNA damage stimulus	796	710	546.85	50	< 1e-30	< 1e-30	8.3e-29
10	GO:0006399	tRNA metabolic process	196	193	134.65	114	8.3e-28	4.9e-05	2.4e-27
11	GO:0006401	RNA catabolic process	238	231	163.51	104	2.8e-29	< 1e-30	1.6e-26
12	GO:0070727	cellular macromolecule localization	1526	1297	1048.36	37	< 1e-30	1.5e-09	3.0e-24
13	GO:0010256	endomembrane system organization	536	464	368.23	143	1.5e-22	0.00367	1.7e-22
14	GO:0032774	RNA biosynthetic process	3821	2911	2625.03	91	< 1e-30	< 1e-30	3.4e-22
15	GO:0045184	establishment of protein localization	1975	1610	1356.83	56	< 1e-30	1.3e-06	3.1e-21
16	GO:0016482	cytoplasmic transport	1243	1058	853.94	51	< 1e-30	6.1e-12	4.9e-20
17	GO:0071826	ribonucleoprotein complex subunit organi...	208	196	142.9	166	7.5e-20	9.7e-19	6.8e-20
18	GO:0044403	symbiosis, encompassing mutualism throug...	845	733	580.52	73	< 1e-30	3.7e-24	1.3e-19
19	GO:0043161	proteasome-mediated ubiquitin-dependent ...	376	348	258.31	100	7.9e-30	7.5e-08	1.7e-19
20	GO:0007264	small GTPase mediated signal transductio...	922	755	633.41	160	9.2e-21	4.4e-07	3.2e-18

Supplementary Figure S4.1 Watt. 2018. TOP.

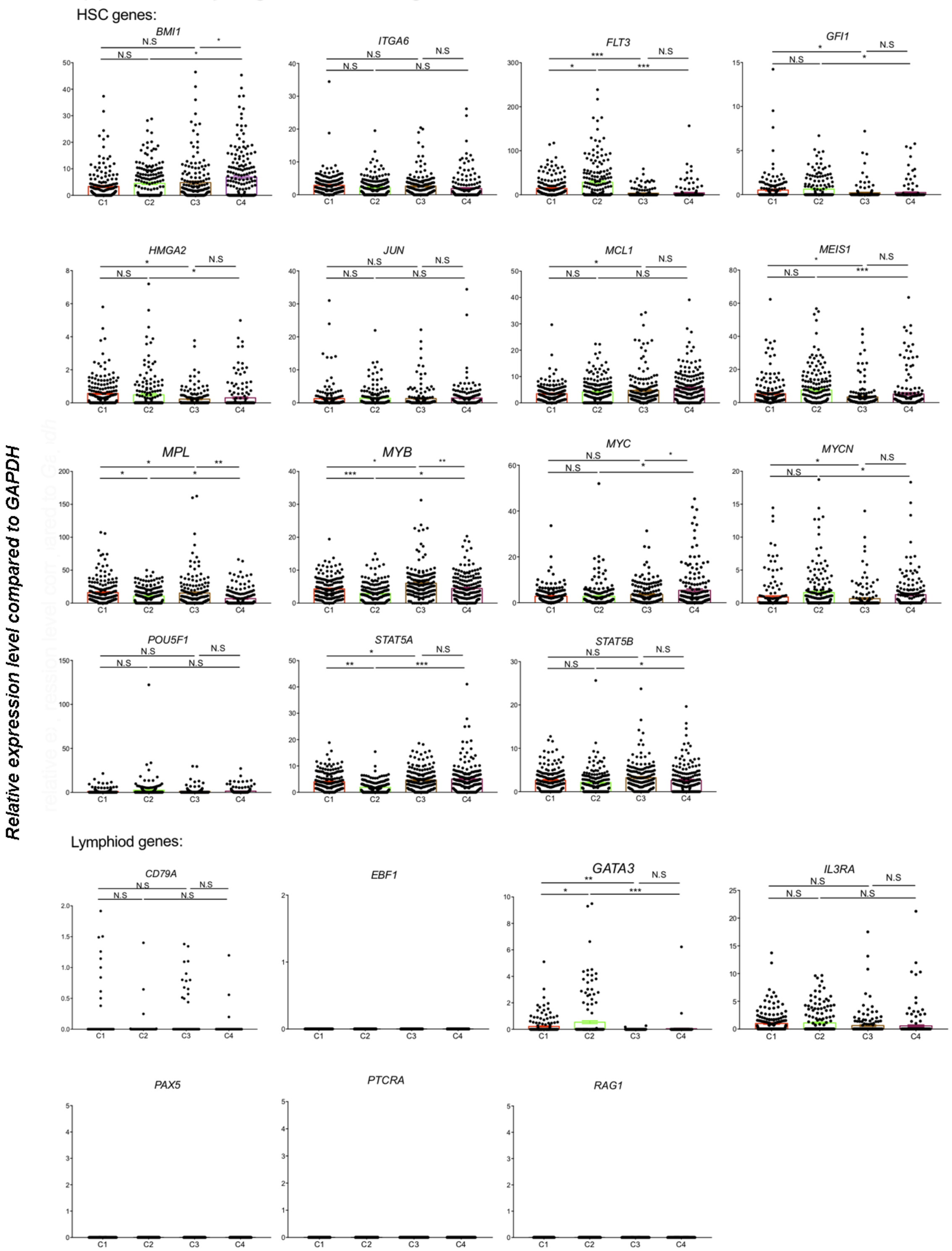

Supplementary Figure S4.2 Watt. 2018. TOP.

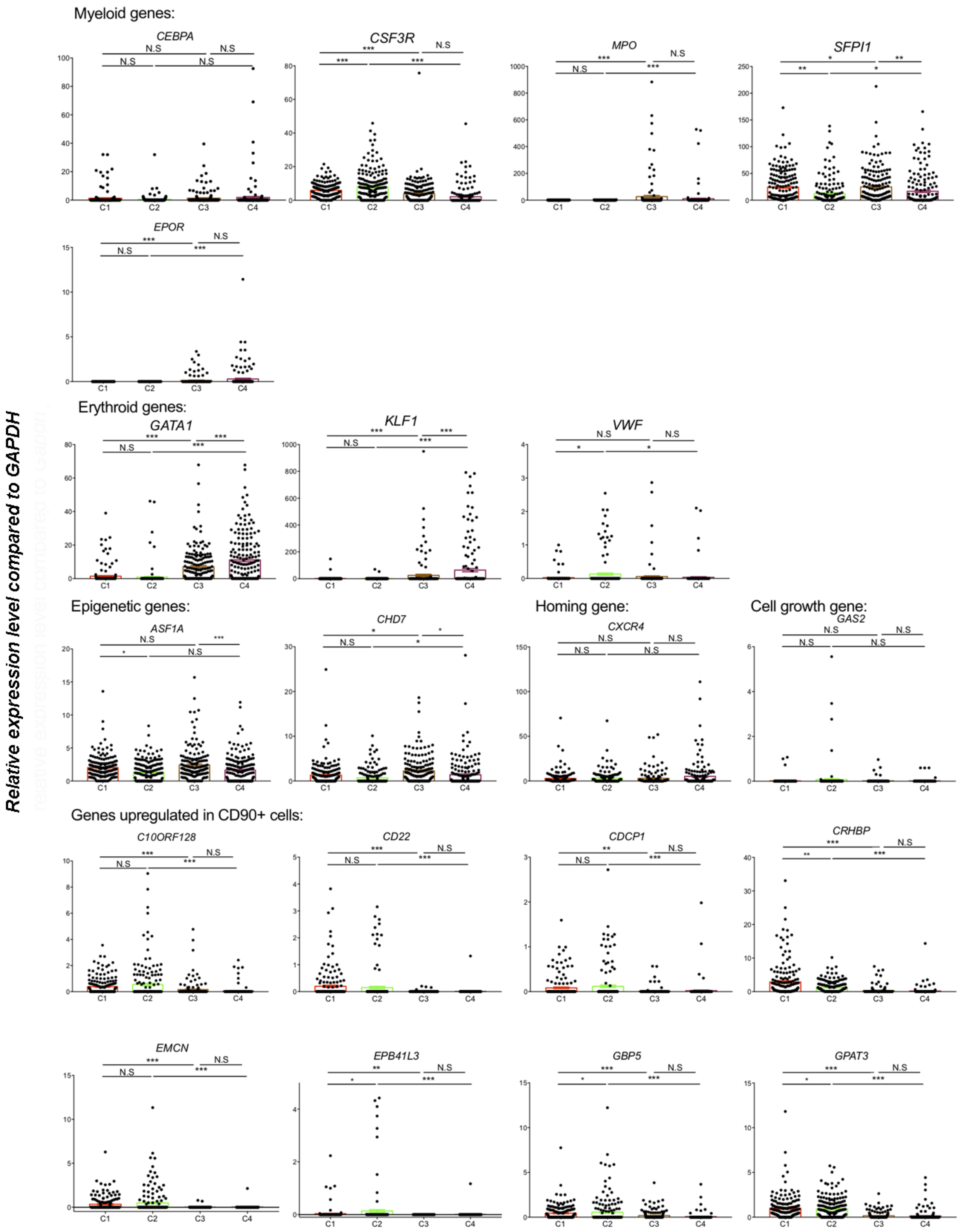

Supplementary Figure S4.3 Watt. 2018. TOP.

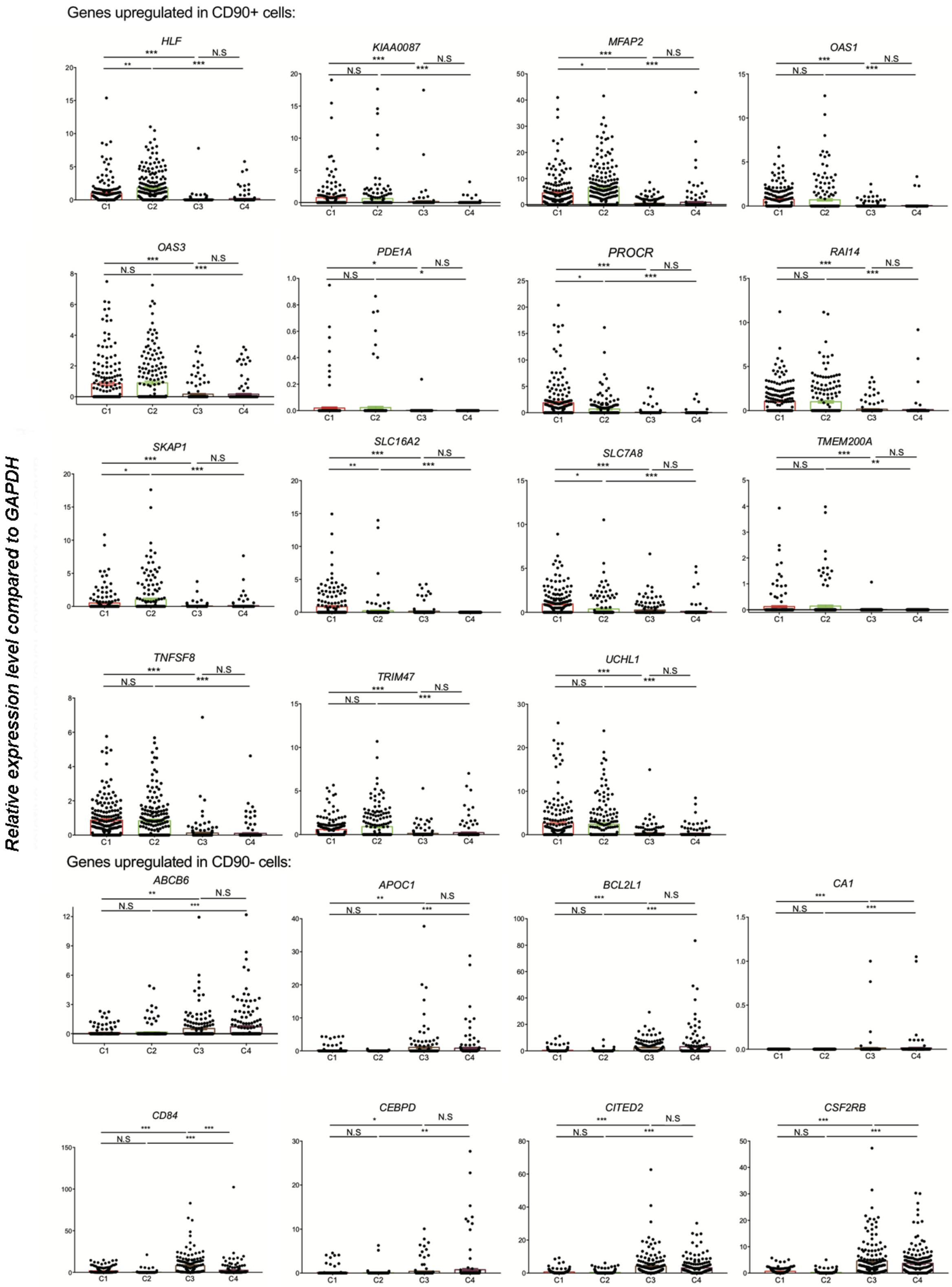

Supplementary Figure S4.4 Watt. TOP.

Genes upregulated in CD90- cells:

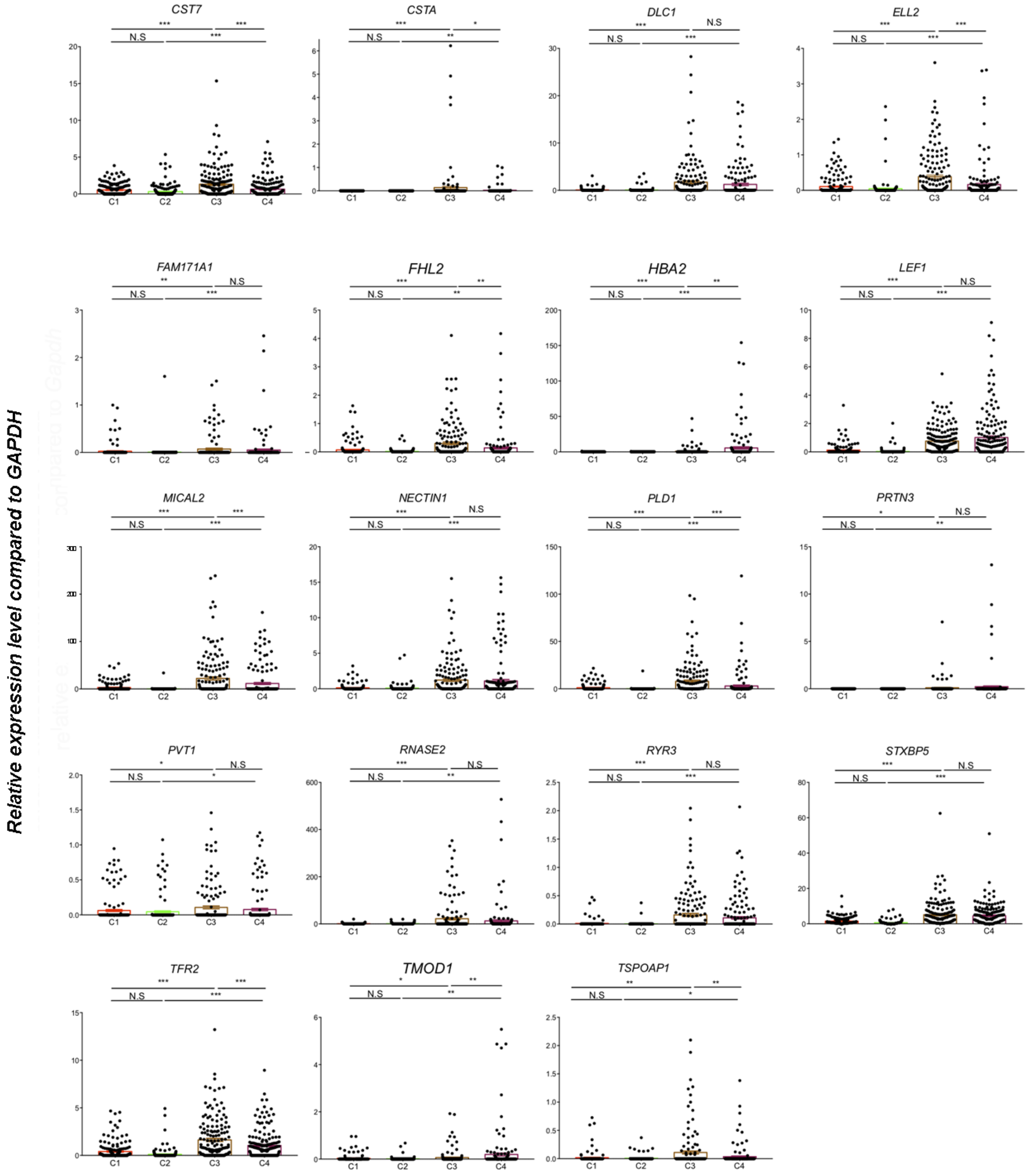

Supplementary Figure S5. Watt. 2018. TOP.

Overnight-cultured UCB CD133+ cells (single live cells)

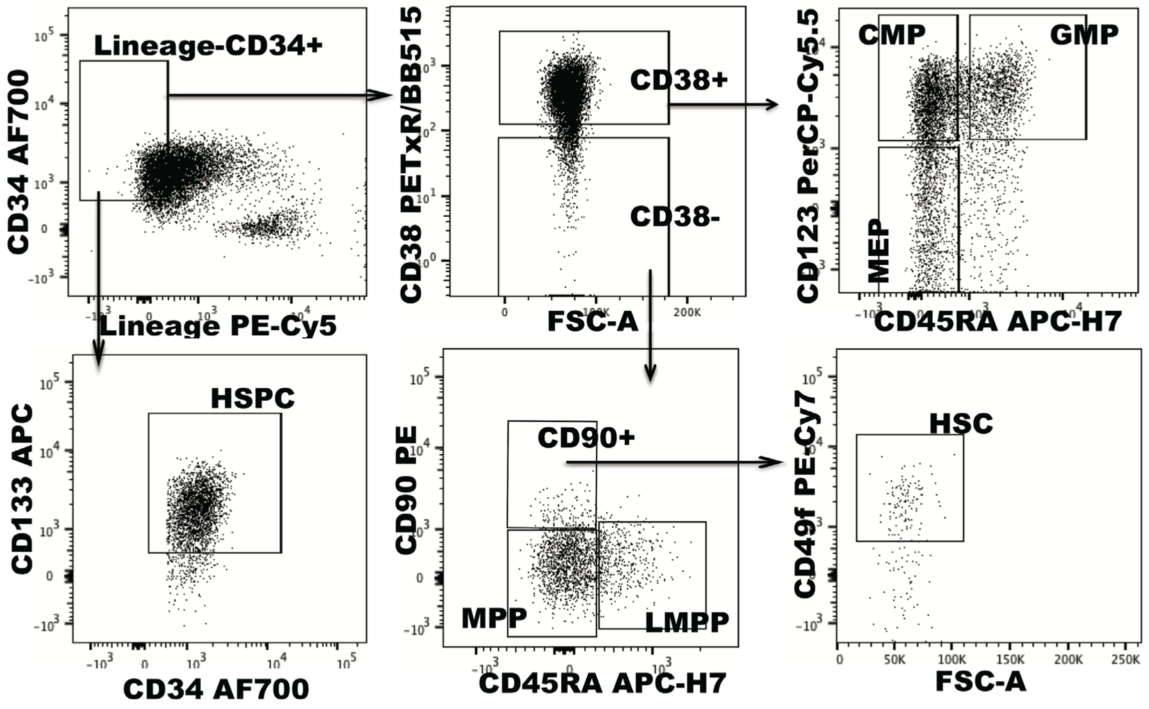

Cytokines-expanded UCB CD133+ cells (single live cells from one well)

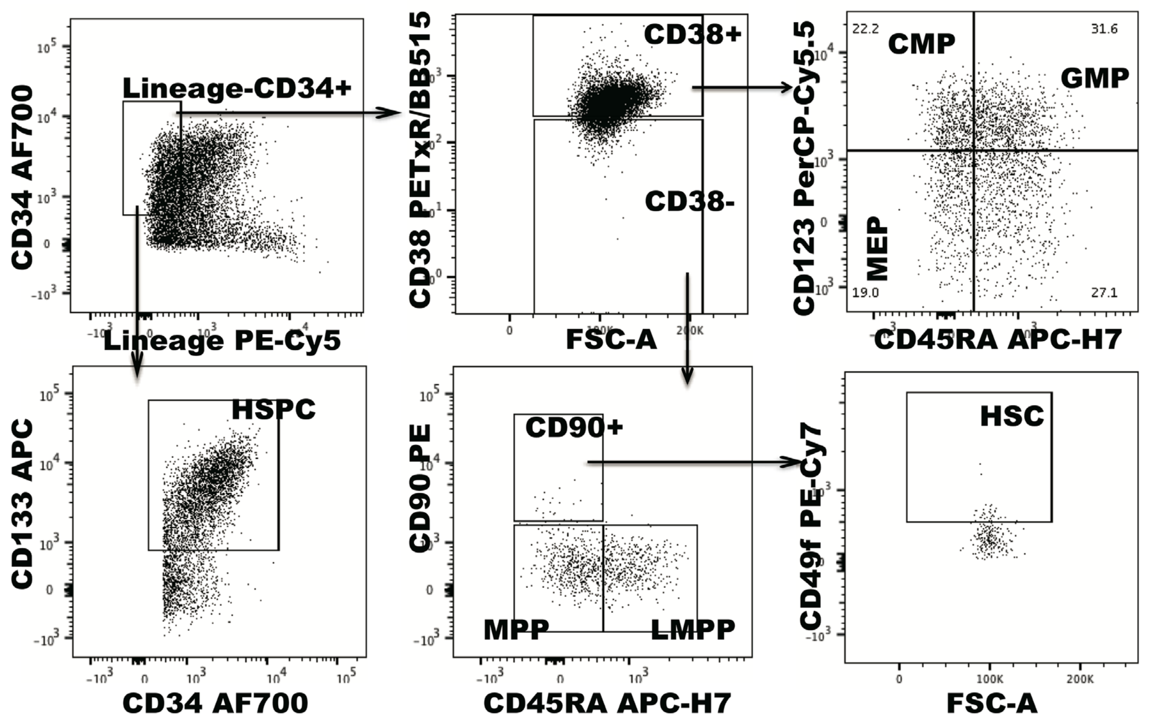

Supplement: Supplementary file 1 — Supplementary Information [file 41598_2019_41803_MOESM1_ESM.pdf]
